# Adherence of popular smoking cessation mobile applications to evidence-based guidelines

**DOI:** 10.1186/s12889-019-7084-7

**Published:** 2019-06-13

**Authors:** Nikita B. Rajani, Dominik Weth, Nikolaos Mastellos, Filippos T. Filippidis

**Affiliations:** 1 Department of Primary Care and Public Health, School of Public Health, Imperial College London, Reynolds Building, St. Dunstan’s Road, London, W6 8RP UK; 2 Department of Strategy and Innovation, MSU Consulting GmbH, Bad Homburg, Germany

**Keywords:** Smoking cessation, Mobile applications, mHealth, Cessation guidelines

## Abstract

**Background:**

Smoking remains one of the major preventable causes of chronic diseases. Considering the promising evidence on the effectiveness of mobile technology for health behaviour change, along with the increasing adoption of smartphones, this review aims to systematically assess the adherence of popular mobile apps for smoking cessation to evidence-based guidelines.

**Methods:**

The United Kingdom Android and iOS markets were searched in February 2018 to identify smoking cessation apps. After screening, 125 Android and 15 iOS apps were tested independently by two reviewers for adherence to the National Institute of Care and Excellence (NICE) Smoking Cessation Guidelines for Self-Help Materials and the Five A Guidelines for Smoking Cessation. Pearson chi square tests were run to examine differences between the two operating systems.

**Results:**

A majority of apps across both operating systems had low adherence (fulfils 1–2 out of 5 guidelines) to the Five A Guidelines (65.7%) and low adherence (fulfils 1–3 out of 9 guidelines) to the NICE Smoking Cessation Guidelines for Self-Help Materials (63.6%). Only 15% of mobile apps provided information about the benefits of nicotine replacement therapy (NRT), and even fewer provided information regarding types of NRT products (7.1%) or how to use them (2.1%). In addition, only a minority of apps arrange follow-up appointments or provide additional support to help smokers quit.

**Conclusion:**

Similar to previous mobile app reviews dating back to 2014, our findings show that most mobile apps do not follow existing smoking cessation treatment guidelines, indicating little change regarding the availability of evidence-based mobile apps for smoking cessation in the UK market. Smokers seeking to quit, tobacco control policy makers and software developers need to work together to develop apps that are in line with the latest clinical guidelines and strategies to maximise effectiveness.

**Electronic supplementary material:**

The online version of this article (10.1186/s12889-019-7084-7) contains supplementary material, which is available to authorized users.

## Background

Despite the well-known health consequences of tobacco, 1.1 billion people in the world still smoke, causing tobacco to be responsible for approximately 7 million deaths globally every year [1]. In the United Kingdom (UK), although smoking prevalence is at an all-time low, smoking remains one of the major preventable causes of chronic diseases [2]. Whilst a majority of smokers want to quit, only 37% of smokers in England attempted to quit in 2014 of which 19% were successfully abstinent for at least four weeks [3]. In terms of long-term success, research shows that only 3–5% of self-quitters remain abstinent after 6–12 months [4]. Mobile applications (apps) have become increasingly popular as a tool to support health behaviour change and health outcomes. According to one study, 400 apps to aid smoking cessation were available in the United States, UK and Australian market in 2015 [5]. With the proliferation of smartphone usage and the booming app markets, this number is likely to have grown.

Mobile phone-based cessation interventions have been found to have a positive impact on cessation outcomes. Considering 6-month cessation outcomes, a systematic review showed that smokers who received support from mobile phone-based interventions were 1.7 times more likely to remain quit compared to those who did not receive such support [6]. Similarly, Zeng et al. (2016) found that individuals who were fully adherent to a mobile app intervention were four times more likely to quit than individuals who were not utilizing the app at two months follow-up [7]. Bricker et al. (2017) also found that the use of a mobile app-based intervention resulted in modest quit rates and higher rates of smoking reduction at 2 months follow-up [8]. Prior studies have found that input from health care professionals and scientists during the development of mobile apps to ensure they are evidence-based is likely to result in apps with more reliable content and of higher quality [9]. Given the promising evidence on the effectiveness of mobile technology, particularly evidence-based mobile apps, and the increasing adoption rates of smartphones, it is important that research systematically examines information presented in these apps.

A number of reviews on smoking cessation apps have been conducted in various markets such as Australia, Portugal, United States, South Korea, and the UK [10–16]. Several of these reviews have investigated the content, quality and adherence to smoking cessation treatment guidelines. The general findings indicate that apps are of poor quality and have low adherence to treatment guidelines. Of these, two reviews investigated the market of mobile apps for smoking cessation in the UK [15, 16]. Both reviews examined the use of behaviour change techniques in mobile apps for smoking cessation available on the UK iOS market in 2012 and 2014. The behaviour change techniques were identified from a prior study examining treatment manuals from stop smoking services in the UK. Both reviews also investigated engagement features (i.e. personalisation, design, dashboards etc.) and ease-of-use features (i.e. aesthetics, minimum text, page names, font size etc.) in the apps.

However, both reviews did not consider apps available on the Android operating system, although the majority of the market is dominated by Android smartphones [17]. Moreover, some studies indicate that there are differences between the users of the two operating systems. According to Smith (2013), individuals with higher socioeconomic status were more likely to own an iPhone whilst the distribution and use of Android smartphones was equal across various levels of income and education [18]. Since the literature also shows that smoking prevalence is four times greater amongst the most disadvantaged compared to the affluent population groups in the UK, and that smokers with lower socioeconomic status are less likely to stop smoking successfully compared to smokers of higher socioeconomic status, it is vital that a review of mobile apps includes those available for Android smartphones [19, 20]. Additionally, the prior reviews examined the adherence of apps to evidence-based techniques based on face-to-face behaviour support manuals from 2008 to 2009, which are no longer up-to-date nor tailored for interventions that do not involve face-to-face contact or personalised interaction.

Due to the constantly evolving nature of the mobile app market, country specific availability of apps and treatment guidelines, it is important to conduct a review of apps currently available to help smokers quit in the UK. It is also critical to gain a better understanding of the content of apps that are being used by the public and whether they are in line with existing evidence and guidelines. Therefore, the main objectives of this research were to explore the price and general functionalities of popular apps for smoking cessation currently available in the mobile app market in the UK, and to investigate the adherence to evidence-based smoking cessation guidelines for self-help materials developed by the National Institute of Care and Excellence (NICE) These guidelines have been developed more recently and are tailored for cessation programmes that smokers can try to use independently to quit smoking or reduce the number of cigarettes they smoke “without the help of health professionals, stop smoking advisors or support groups”, such as mobile apps [21].

## Methods

### Search strategy

Mobile apps available on the UK Android and iOS markets were identified and extracted using the app market Application Programming Interface (API) provided by the software 42matters on 19th February 2018 [22]. The search terms used to identify mobile apps included “stop smoking”, “quit smoking”, and “smoking cessation”. These terms are consistent with prior mobile app reviews on smoking cessation [11–15].

### Preliminary inclusion and exclusion

Upon identification of mobile apps, apps were screened to ensure that only apps which met the inclusion and exclusion criteria were included in the study. During this stage, duplicate apps within each market were eliminated based on identification numbers. With the aim to exclude apps with a very small number of users, apps were further screened for popularity based on two metrics: the number of ratings and the rating itself. Although app stores do not reveal the exact methodology and variables used to rank apps, it is accepted that the rating, number of ratings, downloads and reviews can be used to determine an app’s popularity [23]. Since the Apple Store only includes ratings for apps that have been rated by at least five individual users, the same cut-off point was applied to Android apps; for consistency all Android apps with less than five user ratings were excluded. Additionally, consistent with prior reviews, mobile apps with less than a four-star rating were excluded from both stores. Using popularity as an inclusion or exclusion criteria for mobile app reviews is a common methodology adopted in past studies as it ensures the most widely used and most ‘liked’ apps, which are also the most likely to be downloaded by smokers seeking to quit, are evaluated [23–25]. A study showed that many smokers and drinkers believed taking “other people’s star ratings or reviews of apps into account was vital in guiding their choice” [26]. Further studies have also identified strong correlations between app ratings and number of downloads suggesting that low rated apps are less likely to be downloaded [27, 28].

### Screening process

In the next stage, a more thorough screening process was conducted. All remaining apps were screened based on the description and screenshots provided on the product page in the app stores. Mobile apps that suggested they could help smokers quit smoking were included whilst irrelevant apps were excluded. Apps were considered irrelevant when they had nothing to do with smoking cessation but were still captured by the software due to the inputted search terms. Examples of irrelevant apps include but are not limited to apps that are online vape shops, apps about restaurants or bars that specify in their description that they are non-smoking, apps that use the term smoking as an adjective etc. Apps advertising hypnosis methods to quit smoking were excluded because hypnosis is not an evidence-based strategy for smoking cessation [14, 29]. Other exclusion criteria included apps aiming to help specific patient groups or healthcare professionals and apps that required additional equipment to be fully functional (e.g. smartwatches, DVDs). The inclusion and exclusion criteria used to screen the apps are summarised in Additional file 2: Table S1. Screening was undertaken by two reviewers independently. Results and discrepancies were identified and resolved through discussion between the reviewers. If no agreement could be reached, a third reviewer was consulted to make a final decision.

### Procedure of coding Mobile apps

Each app was reviewed for approximately 30 min on the day of installation and a check for additional notifications delivered by the app was conducted the following day. Mobile apps were coded to identify general functionalities and adherence to smoking cessation guidelines. Similar to screening, apps were tested by two reviewers and a third reviewer was consulted if discrepancies could not be resolved.

#### General functionalities

Each app was assessed for the type of approach or feature it incorporated to aid smokers with quitting. The categories used to classify the mobile apps were initially created by the National Tobacco Cessation Collaborative and have been consistently used in prior mobile app reviews examining smoking cessation apps. Additional file 2: Table S2 displays the various categories along with a brief explanation of what such an app within the category would entail.

#### Smoking Cessation Guidelines

Adherence to smoking cessation guidelines was assessed by evaluating apps against two sets of evidence-based guidelines. Firstly, mobile apps were assessed against the FIVE A’s framework for behaviour change [30]. This framework has been adapted for several health behaviours such as smoking, alcohol consumption and physical activity, and is globally accepted as a guiding tool for health behaviour change interventions.

Mobile apps were also evaluated against the National Institute of Care and Excellence (NICE) guidelines for smoking cessation self-help materials [21]. These are official evidence-based guidelines recommended by the NICE institute in the UK for self-help materials such as mobile apps. The Five A’s framework for behaviour change and the NICE guidelines for smoking cessation self-help materials are presented in Additional file 2: Table S3.

### Data analysis

Descriptive statistics were used to examine the price, ratings, and functionalities of mobile apps, as well as adherence to smoking cessation guidelines. Means and standard deviations for price, user rating and number of ratings were calculated. To facilitate description and comparisons, adherence to Five A’s was arbitrarily classified as none; low (i.e. adherence to 1–2 elements); medium (3–4 elements); and high (5 elements). Adherence to NICE guidelines was classified as none; low (1–3 elements); medium (4–6 elements); and high (7–9 elements). Pearson chi-square tests for independence were used to examine differences between the two operating systems. When frequency counts were less than 5, Fisher’s exact test of independence was used. A significance level of *p* < 0.05 was set to determine statistical significance. All statistical analyses were conducted using STATA 12.1.

## Results

### App search results

The software identified a total of 962 iOS apps and 1551 Android apps as a result of the inputted search terms. After all screening was completed, 15 iOS and 125 Android apps were tested and evaluated against predetermined criteria. In total, 140 mobile apps were evaluated for app functionalities and smoking cessation treatment guidelines. A detailed flowchart summarising the results of the app search and the screening process is included in Additional file 1: Figure S1.

### App functionalities

Absolute and relative frequencies of the general characteristics of mobile apps for smoking cessation across both operating systems are shown in Table 1. The average price, user rating and number of ratings (with respective ranges) are also reported in Table 1. The most common feature across both systems allowed users to track the day until and/or since quitting (86.4%). A large proportion of apps included a calculator function which helped users calculate money saved or health benefits accrued since quitting (80.3%). These two features combined highlight the primary functionalities of smoking cessation apps. On the other hand, game and lung health monitoring features were not popular (11.4 and 0% respectively). Most apps tested in both operating systems were free (85.0%). Only apps that had a rating of 4 or 5 were included in the analysis; therefore, the average user rating for iOS and Android apps was quite similar, 4.6 and 4.4 respectively.

**Table 1:** Overview of Mobile Apps for Smoking Cessation

		Platform		
		iOS (*n* = 15)	Android (*n* = 125)	Both Systems (*n* = 140)
Features of Applications	Calculator	15 (100%)	99 (79.2%)	114 (80.3%)
	Rationing	1 (6.7%)	24 (19.2%)	25 (17.9%)
	Tracker	15 (100%)	106 (84.8%)	121 (86.4%)
	Informational	4 (26. 7%)	18 (14.4%)	22 (15.7%)
	Game	0 (0%)	16 (12.8%)	16 (11.4%)
	Lung Health Monitor	0 (0%)	0 (0%)	0 (0%)
	Other	1 (6.7%)	4 (3.2%)	5 (3.6%)
Cost	Free	14 (93.3%)	105 (84.0%)	119 (85.0%)
	Paid	1 (6.7%)	20 (16.0%)	21 (15%.0)
	Mean Price (£)	1.0 (0.0–0.99)	2.2 (0.0–8.6)	2.1 (0.0–8.6)
Popularity	Mean User Rating	4.6 (4.1–5.0)	4.4 (4.0–5.0)	4.4 (4.0–5.0)
	Mean Number of Ratings	821 (6–6500)	1726 (6–35,045)	1629 (6–35,045)

### Smoking cessation treatment guidelines

Table 2 presents the number and percentage of apps adhering to smoking cessation guidelines. When assessed for adherence to the Five A’s, the majority of mobile apps for smoking cessation ASK users whether or not they smoke cigarettes (84.3%). Several mobile apps ADVISE users to quit smoking (49.3%). This is often done by explicitly asking users to quit for health benefits, or providing encouragement for reaching the decision of quitting. Whilst adherence to these two guidelines (ASK and ADVISE) is relatively high, only a minority of mobile apps ASSESS the readiness of users to quit or ARRANGE a follow-up visit (10.7 and 10.0% respectively). Finally, 32.9% of mobile apps across both operating systems provide some form of assistance to users on how to quit smoking (ASSIST) predominantly by including tracker and calculator features. Based on results from chi-square tests the two operating systems differed in adherence to the ARRANGE guideline (33.3% in iOS vs. 7.2% in Android; *p* = 0.001).

**Table 2:** Number and Percentage of Apps Adhering to Smoking Cessation Guidelines

Smoking Cessation Guidelines		Platform			Chi-Square
		iOS (*n* = 15)	Android (*n* = 125)	Both (*n* = 140)	*P*-Value
Five A Framework	ASK	12 (80.0%)	106 (84.8%)	118 (84.3%)	0.706
	ADVISE	9 (60.0%)	60 (48.0%)	69 (49.3%)	0.380
	ASSESS	3 (20.0%)	12 (9.6%)	15 (10.7%)	0.204
	ASSIST	6 (40.0%)	40 (32.0%)	46 (32.9%)	0.533
	ARRANGE	5 (33.3%)	9 (7.2%)	14 (10.0%)	0.001*
Guidelines for Smoking Cessation Self-Help Materials (NICE)	Harm reduction	4 (26.7%)	17 (13.6%)	21 (15.0%)	0.232
	Benefits of quitting	11 (73.3%)	82 (65.6%)	93 (66.4%)	0.773
	Planning a schedule	7 (46.7%)	31 (24.8%)	38 (27.1%)	0.072
	Strategies to cut down	9 (60.0%)	16 (12.8%)	25 (17.9%)	< 0.001*
	Benefits of NRT	5 (33.3%)	16 (12.8%)	21 (15.0%)	0.035*
	Types of NRT products	3 (20.0%)	7 (5.6%)	10 (7.1%)	0.076
	How to use NRT products	0 (0.0%)	3 (2.4%)	3 (2.1%)	0.999
	Where to get NRT products	0 (0.0%)	3 (2.4%)	3 (2.1%)	0.999
	Where to get further support	1 (6.7%)	10 (8.1%)	11 (7.9%)	0.999

*P-value < 0.05

When assessed for adherence to guidelines for self-help materials devised by NICE, the most common guideline followed by mobile apps across both operating systems was including the information regarding the benefits of quitting (66.4%). However, only a minority of apps provided information regarding the benefits of NRT products (15.0%), the types of products that can be used (7.0%), how to use the products (2.1%) or where to get them (2.1%). Moreover, only 7.9% of mobile apps across both operating systems provided resources or direction to seek further support. Similar to the Five A’s, statistically significant differences between the two operating systems were not found for adherence to most smoking cessation guidelines (*p*-value> 0.05). However, apps in iOS were more likely to provide information on the benefits of NRT (33.3% vs. 12.8%; p-value = 0.035) and information on strategies to cut down smoking (60.0% vs. 12.8%; p-value = 0.000).

Table 3 presents the level of adherence of mobile apps by assessing the number of guidelines met by mobile apps across both operating systems. A majority of apps across both systems had low adherence to the Five A’s (65.7%) as well as low adherence to NICE guidelines for smoking cessation self-help materials (63.6%). Only 4 apps out of 140 had high adherence to the evidence-based treatment guidelines specifically set out by a UK institute.

**Table 3:** Level of Adherence to Smoking Cessation Guidelines

Smoking Cessation Guidelines		Platform		
		iOS (n = 15)	Android (*n* = 125)	Both (*n* = 140)
Five A Framework	None (0)	0 (0.0%)	9 (7.2%)	9 (6.4%)
	Low (1–2)	8 (53.3%)	84 (67.2%)	92 (65.7%)
	Medium (3–4)	6 (40.0%)	30 (24.0%)	36 (25.7%)
	High (5)	1 (6.7%)	2 (1.6%)	3 (2.1%)
NICE Guidelines for Smoking Cessation Self-Help Materials	None (0)	2 (13.3%)	31 (24.8%)	33 (23.6%)
	Low (1–3)	8 (53.3%)	81 (64.8%)	89 (63.6%)
	Medium (4–6)	4 (26.7%)	10 (8.0%)	14 (10.0%)
	High (7–9)	1 (6.7%)	3 (2.4%)	4 (2.9%)

## Discussion

The current study reviewing 140 mobile apps expands on the current knowledge of available apps for smoking cessation in the UK. The main finding was that most mobile apps had low adherence to Five A’s and NICE guidelines and only had selected features among those recommended in the guidelines. This finding is consistent with the findings of mobile app reviews conducted outside of the UK market which found low adherence to country-specific smoking cessation guidelines [11–14] Our study found that most apps available on the UK market allowed users to track the day until and/or since quitting and to calculate the money saved or health benefits accrued since quitting. These were the two most popular features or functionalities of mobile apps across both operating systems which is consistent with prior mobile app reviews [11–14]. Such features allow users to track their progress and visualise the benefits of their behaviour change, which in turn may function as a motivating factor or incentive to remain quit.

When assessing the apps against the Five A’s framework for interventions, two thirds of apps across both operating systems had a low level of adherence. Only a minority of mobile apps assessed the readiness of users to quit or arranged a follow-up visit. It may seem unnecessary for apps to assess readiness to quit if users of the app have already made the decision to quit. However, research shows that interventions which do not address readiness to quit fail to positively reinforce a crucial decision, are unable to address barriers to quitting and are less likely to help smokers work towards a higher level of readiness for change [31]. Similarly, only 2.9% of all mobile apps across both operating systems had high adherence to NICE guidelines for smoking cessation self-help materials. Although most apps highlighted the benefits of quitting, very few discussed NRT treatment or products. The benefit of using NRT to quit smoking is well-established, and clinically recommended by NICE as the first line of treatment for individuals seeking pharmacological help [32]. The use of NRT as a type of cessation method increase quitting rates by 50–60% [32]. Since NRT is proven to be helpful, omitting this from smoking cessation interventions may limit their effectiveness and ability to help smokers quit. Funding streams which encourage collaboration between health researchers and app developers may facilitate the inclusion of key public health recommendations into new mobile apps. Moreover, academics interested in the development of health apps but lack funding and dissemination expertise, could try to establish industry partnerships in the early stages to take advantage of experienced help in the commercialisation and wide dissemination of apps [33]. As suggested by Arigo et al. (2019), behavioural medicine professional organisations can play a vital role in enabling such partnerships and collaborations [33].

The use of low-quality mobile apps can reduce users’ confidence in quitting or continuing a quit attempt. This can have negative consequences as it can hinder users from pursuing better quality smoking cessation interventions, or hinder quit attempts altogether. Therefore, it is vital for mobile app developers to work together with smoking cessation and public health experts to ensure that apps are developed in line with treatment guidelines to achieve the highest possible impact. The few apps that exhibited high adherence to evidence-based guidelines were not necessarily the most popular, highlighting the need to promote apps that adhere to guidelines and ensure that they remain visible and accessible in the volatile mobile app market. We could not differentiate between apps developed with input from scientists and those without, hence we haven’t been able to compare them. Future research could investigate the development process of such apps to better understand which aspects (e.g. engagement features, aesthetics etc.) are being prioritised by developers instead of treatment guidelines. Solutions such as the digital assessment tool offered by the National Health Service (NHS) Apps Library may contribute to the development of more evidence-based, clinically safe and secure apps [34].

Technology-enabled interventions provide a relatively low-cost method of reaching a large number of people, therefore it is important that apps that adhere to treatment guidelines are offered and disseminated to smokers seeking to quit. Past studies have shown that mobile interventions have helped participants with lower socioeconomic status more than those with higher socioeconomic status [19]. Thus, developing and promoting effective smoking cessation mobile apps may attenuate health disparities in the prevalence of smoking and smoking cessation, especially at a time when the proportion of smokers who use evidence-based cessation assistance is declining [35].

Although most apps from both operating systems did not arrange additional support for the user, statistically significant differences were detected between the two operating systems. Similarly, the two operating systems differed with regard to provision of strategies to cut down smoking and benefits of NRT products. However, it is important to acknowledge that the limited sample size of iOS apps assessed in our review limits the generalizability of these findings. A recent study found a lower rate of medication use among smokers who used the iOS version of a smoking cessation app compared to Android users [36]. Although this finding does not provide an explanation for why iOS apps were more likely to include information about NRT, it shows that iOS and Android smartphone users can differ in ways that could affect the use and effectiveness of the app, in turn prompting developers to alter content based on the type of user (iOS or Android). Future research could further investigate content differences between operating systems and consider operating systems as a variable when comparing the effectiveness of mobile apps.

Our mobile app review has several strengths. One of the main advantages is that it focuses on apps available on the UK market, which has been sparsely investigated in the literature. It also investigates apps available on both iOS and Android and does not limit itself to free apps which ensures that the findings are representative of the whole app market. Furthermore, as the app data was captured in February 2018, the results provide a more accurate and updated overview of the market compared to earlier analyses. However, we did not assess apps that had lower than a 4-star rating and fewer than 5 individual ratings. Particularly for apps from the Apple store, this resulted in the exclusion of a large number of apps. Another mobile app review found more than half of the iOS apps tested in their study had no rating [37]. This may limit the range of apps that smokers using iOS are likely to download, but it is unlikely to have impacted the conclusions of our review, as our interest was in popular apps. Although excluded apps may differ from those included in the review, it is likely that they are being used by a relatively small proportion of smokers who are trying to quit. Future reviews could include apps with a lower user rating and perform further analyses to compare user ratings of evidence-based and non-evidence-based apps. A better understanding of whether apps with high adherence to cessation guidelines were developed with researchers could encourage collaboration between health researchers and app developers. We were also not able to assess apps that may offer support for smoking cessation but were not captured by the software used through the input of specific, commonly used, search terms. All mobile apps were reviewed for approximately 30 min on the day of installation and a few minutes the following day; certain app functionalities which are only visible or activated upon long-term use would not have been recorded.

## Conclusion

Our research provides a comprehensive overview of the mobile app market for smoking cessation in the UK in early 2018. Our findings show that adherence to smoking cessation treatment guidelines was generally low, which may potentially have a negative impact on the apps’ effectiveness. Increasing adherence to guidelines could maximise the impact of such services on smoking cessation at a relatively low cost. Our research can inform choices of smokers attempting to quit, software developers, policy makers and tobacco control professionals. Stronger collaboration between experts in smoking cessation and software developers is essential to produce more effective mobile applications in the future.

## Additional files

Additional file 1: **Figure S1.** Flowchart of Results from App Search, Preliminary Inclusion & Exclusion, Screening, Final App Pool. **Figure S1.** visually displays the results of the key stages of the mobile app review. The key stages include app search, preliminary inclusion and exclusion, screening and final app pool. (DOCX 103 kb)

Additional file 2: **Table S1.** Inclusion and Exclusion Criteria, **Table S2.** Classification of Mobile Apps, **Table S3.** Evidence-Based Smoking Cessation Guidelines. **Table S1.** displays the inclusion and exclusion criteria which were used during the screening phase of the mobile app review. **Table S2.** displays the key functionalities and features of mobile apps. These categories were used to classify mobile apps during the testing phase of the mobile app review. **Table S3.** displays the Five A guidelines for smoking cessation and NICE guidelines for smoking cessation self-help materials. Both frameworks were used during the testing phase of the mobile app review to assess level of adherence to evidence-based guidelines. (DOCX 17 kb)

## Data Availability

Data on available mobile apps was obtained and is available on https://42matters.com/.

## Abbreviations

API: Application Programming Interface
NHS: National Health Service
NICE: National Institute of Care and Excellence
NRT: Nicotine Replacement Therapy
UK: United Kingdom
