# Supplementary material for: Adherence of popular smoking cessation mobile applications to evidence-based guidelines
Source: BMC Public Health. 2019 Jun 13;19:743. doi: 10.1186/s12889-019-7084-7 (PMC6567534; doi:10.1186/s12889-019-7084-7)
Supplement: Supplementary file 1 — Figure S1. Flowchart of Results from App Search, Preliminary Inclusion & Exclusion, Screening, Final App Pool. Figure S1. visually displays the results of the key stages of the mobile app review. The key stages include app search, preliminary inclusion and exclusion, screening and final app pool. (DOCX 103 kb) [file 12889_2019_7084_MOESM1_ESM.docx]

Figure S1. Flowchart of Results from App Search, Preliminary Inclusion & Exclusion, Screening, Final App Pool


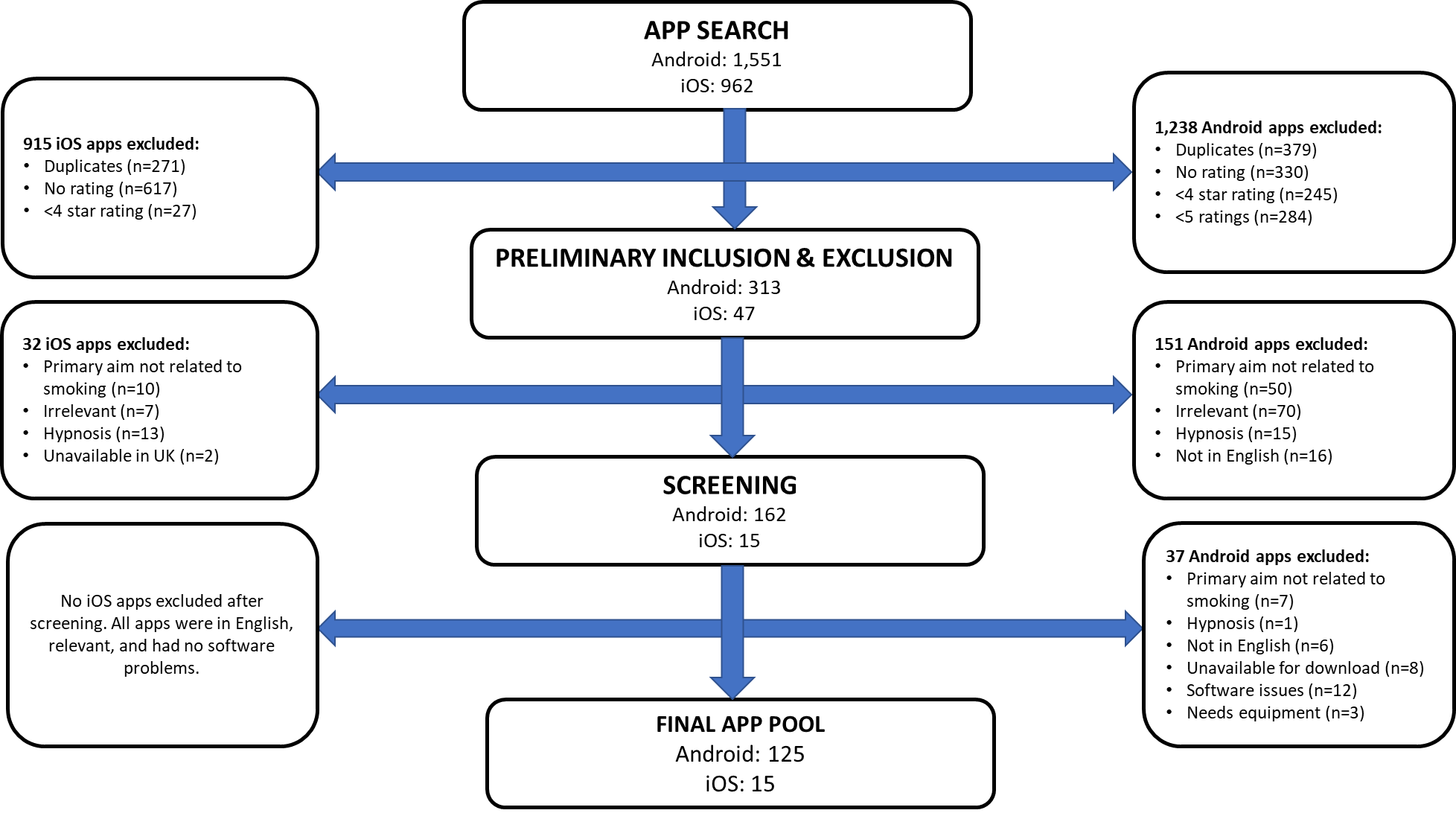


*Figure S1 visually displays the results of the key stages of the mobile app review. Using the software 42matters and the input of relevant search terms, 1,551 Android and 962 iOS apps were identified. After preliminary inclusion and exclusion criteria were applied, 313 Android and 47 iOS apps remained. Further screening based on evaluation of the app product page (on the Google Play or Apple store) resulted in 162 Android and 15 iOS apps. Finally, further exclusion of apps upon download and during the coding procedure resulted in a total app pool of 125 Android and 15 iOS apps.*
