# Supplementary material for: Adherence of popular smoking cessation mobile applications to evidence-based guidelines
Source: BMC Public Health. 2019 Jun 13;19:743. doi: 10.1186/s12889-019-7084-7 (PMC6567534; doi:10.1186/s12889-019-7084-7)
Supplement: Supplementary file 2 — Table S1. Inclusion and Exclusion Criteria, Table S2. Classification of Mobile Apps, Table S3. Evidence-Based Smoking Cessation Guidelines. Table S1. displays the inclusion and exclusion criteria which were used during the screening phase of the mobile app review. Table S2. displays the key functionalities and features of mobile apps. These categories were used to classify mobile apps during the testing phase of the mobile app review. Table S3. displays the Five A guidelines for smoking cessation and NICE guidelines for smoking cessation self-help materials. Both frameworks were used during the testing phase of the mobile app review to assess level of adherence to evidence-based guidelines. (DOCX 17 kb) [file 12889_2019_7084_MOESM2_ESM.docx]

**Additional file 2**

Table S1. Inclusion and Exclusion Criteria

| **Inclusion Criteria** | **Exclusion Criteria** |
| --- | --- |
| Primary aim of the app is to help smokers quit or reduce smoking. App must clearly state that this is its main purpose | App claims it can be used to help quit smoking but this is not the primary aim of the app |
| App is available in the UK (i.e. no country restriction upon download) | App is not in the English language |
| Apps that are fully functional without any major software issues (e.g. crashes upon download or use, videos and features do not load) | App is not available in the UK |
| App targets the general public | App has less than 5 user ratings |
|  | App has less than a 4-star rating |
|  | Hypnosis apps |
|  | App is designed for healthcare professionals or specific patient groups |
|  | App targets substances other than nicotine or other forms of smoking (e.g. marijuana) |
|  | App requires compatibility with other devices or products (e.g. smartwatch, DVDs) |

Table S2. Classification of Mobile Apps

| **Type of App** | **Description** |
| --- | --- |
| Tracker | The app tracks the number of days elapsed since the user quit smoking and/or the number of days until the user’s quit date |
| Calculator | The app primarily calculates the amount of money a smoker saves by not smoking and/or the health benefits attained by not smoking |
| Rationing | The app prompts the user to limit the number of cigarettes smoked and/or how often the user can smoke a cigarette (e.g. providing time limits) |
| Informational | The app provides information in the form of text and images to provide the user with knowledge on various aspects of smoking cessation |
| Game | The app takes the form of a game to help users quit |
| Lung Health Monitor | The app measures and tracks the user’s lung function and health |
| Other | Any other approaches/features that have not been described above |

Table S3. Evidence-Based Smoking Cessation Guidelines

| **Five A’s Guidelines for Smoking Cessation** | |
| --- | --- |
| ASK | The mobile app asks the user whether or not they smoke cigarettes and/or use other tobacco products |
| ADVISE | The mobile app persuades and advises all tobacco users to quit |
| ASSESS | The mobile app assesses the user’s readiness to make a quit attempt. For example, the app can do this by asking questions related to importance or quitting and a self-efficacy. |
| ASSIST | The mobile app assists or helps the user quit. It can do this in various ways: helping create a quit plan, providing counselling, providing support, recommending medications etc. |
| ARRANGE | The mobile app arranges follow-up contact with the user or provides referral to specialist support. |
| **Smoking Cessation Guidelines for Self-Help Materials (NICE Institute)** | |
| Harm reduction | Details about harm reduction (e.g. cutting down before stopping, reduction methods, abstain) are provided |
| Benefits of quitting | An emphasis on the fact that stopping smoking will improve health far more than continuing to smoke, even at a reduced rate (e.g. reduces risk of cancer, COPD, CVD etc.) is evident |
| Planning a schedule | Advice on how to plan a schedule (e.g. set a quit date, schedule on cutting down) is provided |
| Strategies to cut down | Advice on strategies to cut down and gradually stop or reduce the amount they smoke is provided |
| Benefits of nicotine-replacement therapy | Benefits of using licensed nicotine-containing products to reduce the harm from smoking (e.g. safe, effective) is provided |
| Types of nicotine-replacement therapy | Information on the type of licensed nicotine-containing products available is provided |
| How to use nicotine-replacement therapy | Information on how to use licensed nicotine-containing products effectively to manage the cravings, mood swings and other effects of nicotine dependency and to prevent relapse is provided |
| Where to get nicotine-replacement therapy | Information on where licensed nicotine-containing products can be purchased and who is able to supply or prescribe them is provided |
| Further support | Where to get further support (e.g. additional websites, clinics etc.) is provided |
